# Successful Outcome of Chronic Intrahepatic Cholestasis in an Adult Patient with Sickle Cell/**β** ^**+**^ Thalassemia

**DOI:** 10.1155/2014/213631

**Published:** 2014-02-09

**Authors:** Efthymia Vlachaki, Panagiotis Andreadis, Nikolaos Neokleous, Aleka Agapidou, Evaggelia Vetsiou, Panagiotis Katsinelos, Panagiota Boura

**Affiliations:** ^1^Adults Thalassemia Unit, Second Medical Clinic, Aristotle University of Thessaloniki, Ippokration Hospital, Konstantinoupoleos 49, 54642 Thessaloniki, Greece; ^2^Department of Gastroenterology, Second Medical Clinic, Aristotle University of Thessaloniki, Ippokration Hospital, Konstantinoupoleos 49, 54642 Thessaloniki, Greece

## Abstract

Sickle cell/*β* ^+^ thalassemia (Hb S/*β* ^+^thal) is considered as a variant form of sickle cell disease. Acute episodes of vasoocclusive pain crisis are characteristic for sickle cell disorders and may be complicated by an acute or chronic life-threatening organ dysfunction. Chronic intrahepatic cholestasis is a rare and severe complication in sickle cell disease, characterized by marked hyperbilirubinemia and acute hepatic failure with an often fatal course. Despite the fact that patients with Hb S/*β* ^+^thal usually have a mild type of disease, herein we describe an interesting case of chronic intrahepatic cholestasis with successful outcome in an adult patient with Hb S/*β* ^+^thal.

## 1. Introduction

Sickle cell/*β* ^+^ thalassemia (Hb S/*β* ^+^thal) is considered as a variant form of sickle cell disease (SCD). This condition is an inherited hemolytic anemia, associated with multiple acute and chronic complications such as painful vasoocclusive events, cerebral vasculopathy, priapism, liver, spleen, and renal and or lung disease. Chronic intrahepatic cholestasis is a rare and severe complication in sickle cell disease, characterized by marked hyperbilirubinemia and acute hepatic failure with an often fatal course. Despite the fact that patients with Hb S/*β* ^+^thal usually have a mild type of disease, the low values of Hb A and Hb F might be associated sickle cell disease crises [1, 2].

We report an interesting case of chronic intrahepatic cholestasis with successful outcome in an adult patient with Hb S/*β* ^+^thal. The patient gave consent to the publication of this report.

## 2. Case Report

A 37 year Caucasian old male patient with known history of sickle cell disease and beta thalassemia (Hb S/*β* ^+^thal) was attended to our outpatient clinic with a presenting symptom of worsening jaundice. Patient mentioned that he has stopped his regular schedule of exchange transfusions, which for the time being was inevitably his treatment regimen due to his refusal to continue hydroxycarbamide in order to procreate.

The general condition of the patient was good. Physical examination was normal except the above-mentioned skin scleral jaundice and a palpable liver (4 cm below the costal margin) with no sign of pain. According to his past medical history, he had splenectomy at the age of 13 due to entrapment, cholecystectomy, right shin metaphysis osteomyelitis, and bilateral aseptic (avascular) hip necrosis. Interestingly, in the last two years the patient also suffered two recurrent episodes of painless cholestasis which resolved with exchange transfusions and hydroxycarbamide at 20 mg/kg per day (maximum tolerated dose). It has to be mentioned that the recurrent episodes happened when the patient was not adhering to the therapeutic schedule and his hemoglobin S was greater than 30%.

Blood tests showed that hyperbilirubinemia was 46.8 mg/dL and direct bilirubin 18.68 mg/dL. His white blood count (WBC) was 26400 cells/*μ*L, due to splenectomy and severe condition, hemoglobin (Hb) was 7.9 g/dL, and reticulocytes were 28.2%. High-performance liquid chromatography (HPLC) revealed HbS 74.5%, HbF 9.4% and HbA_2_ 6.9%. Liver function tests showed AST 303 U/mL, ALT 108 U/mL, ALP 150 U/mL, and LDH 830 U/mL. Prothrombin time (PT) was 14.9 sec, INR 1.4, and activated partial thromboplastin time (APTT) 38.9 sec. Ferritin level was 350 ng/dL. Differential diagnosis included viral hepatitis, cholecystitis, choledocholithiasis, and biliary duct obstruction, auto immune diseases, Wilson's disease, and Gilbert's syndrome which were all excluded [3].

**Figure 1:**
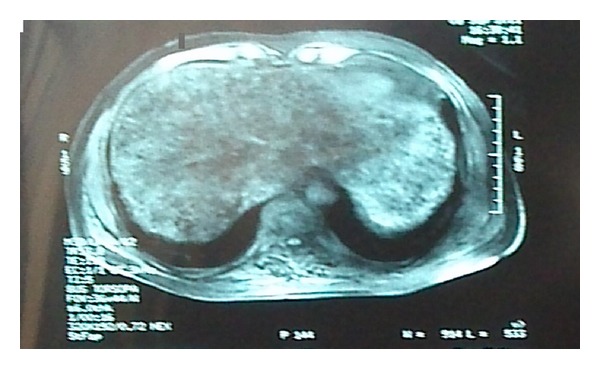
MRI depicting intrahepatic bile duct dilation.

Patient's condition was attributed to chronic intrahepatic cholestasis. Magnetic resonance imaging (MRI) revealed intrahepatic bile duct dilation (Figure 1). Despite documented cases of complications following liver biopsy [4], the patient underwent biopsy which confirmed the cholestasis. He was treated as an outpatient with repeated sessions of exchange blood transfusions on alternate days for two weeks resulted in decrease of both his bilirubin to 8.9 mg/dL and his HbS to 45% and HbA 45%, while his laboratory findings returned to baseline. After his condition was stable, he returned to regular exchange transfusions every 21 days for six months. Thereafter, sperm cryopreservation was performed and he started hydroxycarbamide (20 mg/kg) and regular exchange transfusions for life time. Three years after the episode our patient remains asymptomatic still on regular exchange blood transfusions and hydroxycarbamide.

## 3. Discussion

Sickle cell disease (SCD) is a hemoglobin (Hb) disorder in which substitution of valine for glutamic acid in position 6 of the *β*-globin chain results in an abnormal form of hemoglobin (HbS). SCD refers to all genotypes containing at least one sickle gene, including homozygous HbSS and compound heterozygotes for HbS and C (HbSC) or hemoglobin S and *β* thalassemia (Hb S/*β*°thal or Hb S/*β* ^+^thal) [1, 5].

When this modified hemoglobin is deoxygenated in tissue, it polymerizes and forms rigid aggregates within the red cell which acquire a sickle shape resulting in a loss of flexibility and microvascular occlusion. Acute episodes of vasoocclusive pain crisis are characteristic for sickle cell disorders and may be complicated by an acute, life-threatening organ dysfunction, such as acute chest syndrome, aplastic events, splenic sequestration, and hepatic sequestration with liver dysfunction. The acute dysfunction of the lung or liver has been attributed to sequestration of sickled cells causing microvascular occlusion and tissue ischemia, which may lead to acute organ failure and death [2, 5].

Though hepatic disease is common in sickle cell disease [6], there have been only few similar cases of chronic intrahepatic cholestasis in patients with sickle cell disease and beta thalassemia in contrast with severe painful clinical syndromes like acute hepatic crisis and intrahepatic cholestasis [2, 3, 6–9].

Theoretically, the patients with Hb S/*β* ^+^thal are at low risk to present severe complications. The very low values of Hb A and Hb F in our patient suggested that they might be more susceptible to sickle cell crisis. Genetic markers that affect the level of HbF in Greek origin patients with Hb S/*β* ^+^thal should be studied [10].

Furthermore due to the scarcity of cases, there are no specific diagnostic or therapeutic guidelines making treatment choices and goals difficult to set. It is of great interest that our patient suffered from cholestasis in adulthood compared to other cases that the diagnosis was made at teenagers or young adults. In addition, despite of the risks of liver biopsy in acute hepatic crisis, in this case, the patient underwent successful liver biopsy, after the initiation of exchange transfusion, that finally set the diagnosis. The initial choice of exchange transfusions every 21 days for six months depended upon the patient's desire to semen cryopreservation, because hydroxycarbamide, an S-phase agent that inhibits DNA replication, could potentially have adverse effects on both fertility and pregnancy outcomes. An exhaustive summary on the toxicity of hydroxycarbamide did not document known effects on either fertility or pregnancy in humans. However, a recent publication reported abnormal sperm parameters in adult males with SCD on hydroxycarbamide therapy, which could cause either infertility or teratogenicity [11].

After sperm cryopreservation was performed, our patient was put on hydroxycarbamide (20 mg/kg) and regular exchange transfusions for life time. The combination treatment was chosen because the efficacy of hydroxycarbamide in the treatment of chronic hepatic cholestasis or hepatomegaly has been described only in one case report. In the meantime future studies using hydroxycarbamide to prevent organ dysfunction in SCD are currently under investigation.
